# A pilot feasibility randomized controlled trial on combining mind-body physical exercise, cognitive training, and nurse-led risk factor modification to reduce cognitive decline among older adults with mild cognitive impairment in primary care

**DOI:** 10.7717/peerj.9845

**Published:** 2020-09-07

**Authors:** Zijun Xu, Dexing Zhang, Allen T.C. Lee, Regina W.S. Sit, Carmen Wong, Eric K.P. Lee, Benjamin H.K. Yip, Jennifer Y.S. Tiu, Linda C.W. Lam, Samuel Y.S. Wong

**Affiliations:** 1 Division of Family Medicine and Primary Health Care, JC School of Public Health and Primary Care, Chinese University of Hong Kong, Hong Kong, China; 2 Department of Psychiatry, Faculty of Medicine, Chinese University of Hong Kong, Hong Kong, China

**Keywords:** Dementia prevention, Mild cognitive impairment, Primary care, Multi-component intervention

## Abstract

**Objectives:**

To examine the feasibility and preliminary effectiveness of (1) combining cognitive training, mind-body physical exercise, and nurse-led risk factor modification (CPR), (2) nurse-led risk factor modification (RFM), and (3) health advice (HA) on reducing cognitive decline among older adults with mild cognitive impairment (MCI).

**Methods:**

It was a 3-arm open-labeled pilot randomized controlled trial in the primary care setting in Hong Kong. Nineteen older adults with MCI were randomized to either CPR (*n* = 6), RFM (*n* = 7), or HA (*n* = 6) for 6 months. The primary outcome was the feasibility of the study. Secondary outcomes included the Alzheimer’s Disease Assessment Scale-Cognitive Subscale (ADAS-Cog), the Montreal Cognitive Assessment Hong Kong version (HK-MoCA), the Clinical Dementia Rating (CDR), the Disability Assessment for Dementia (DAD), quality of life, depression, anxiety, physical activity, health service utilization, and diet.

**Results:**

Nineteen out the 98 potential patients were recruited, with a recruitment rate of 19% (95% CI [12–29]%, *P* = 0.243). The adherence rate of risk factor modification was 89% (95% CI [65–98]%, *P* = 0.139) for CPR group and 86% (95% CI [63–96]%, *P* = 0.182) for RFM group. In the CPR group, 53% (95% CI [36–70]%, *P* = 0.038) of the Tai Chi exercise sessions and 54% (95% CI [37–71]%, *P* = 0.051) of cognitive sessions were completed. The overall dropout rate was 11% (95% CI [2–34]%, *P* = 0.456). Significant within group changes were observed in HK-MoCA in RFM (4.50 ± 2.59, *P* = 0.008), cost of health service utilization in CPR (−4000, quartiles: −6800 to −200, *P* = 0.043), fish and seafood in HA (−1.10 ± 1.02, *P* = 0.047), and sugar in HA (2.69 ± 1.80, *P* = 0.015). Group × time interactions were noted on HK-MoCA favoring the RFM group (*P* = 0.000), DAD score favoring CPR group (*P* = 0.027), GAS-20 favoring CPR group (*P* = 0.026), number of servings of fish and seafood (*P* = 0.004), and sugar (*P* < 0.001) ate per day.

**Conclusions:**

In this pilot study, RFM and the multi-domain approach CPR were feasible and had preliminary beneficial effects in older adults with MCI in primary care setting in Hong Kong.

**Trial registration:**

Chinese Clinical Trial Registry (ChiCTR1800015324).

## Introduction

Mild cognitive impairment (MCI) is a transitional state between normal aging process and dementia, characterizing elderly people with cognitive, functional, and memory impairment (Gauthier et al., 2006; Palmer, Fratiglioni & Winblad, 2003). It was reported that about 10% of MCI patients would progress to dementia each year (Behrman, Valkanova & Allan, 2017). Dementia is highly disabling and causes heavy burden to families and society (Castro et al., 2010). Therefore, early diagnosis and intervention, aiming at delaying or even reversing the progress of the disease, has become a major public health priority.

Controlling related risk factors in MCI patients can effectively delay the occurrence and development of dementia (Sanford, 2017). Factors including hypertension, type 2 diabetes mellitus, physical inactivity, obesity, alcohol drinking, smoking, unhealthy diet, and depression were all shown to be associated with dementia (Baumgart et al., 2015). Recent studies showed that aerobic exercise, mind body exercise (Tai Chi), mindfulness, or cognitive-based intervention could improve global cognitive ability and memory in MCI patients (Fitzpatrick-Lewis et al., 2015; Klainin-Yobas et al., 2019; Zheng et al., 2017). In order to modify the multiple risk factors of MCI simultaneously, multicomponent interventions are needed (Olanrewaju et al., 2015; Richard, Moll van Charante & Van Gool, 2012), but relevant studies are limited.

A recent large randomized controlled trial, the Finnish Geriatric Intervention Study to Prevent Cognitive Impairment and Disability (FINGER), was conducted to evaluate the effect of a 2-year multi-domain intervention of diet, exercise, cognitive training, and vascular risk monitoring on cognitive function elderly people at risk of dementia (Ngandu et al., 2015). Significant intervention effects were noted on overall cognition, executive functioning, and processing speed. In Hong Kong, a 1-year randomized controlled trial comparing the integrated cognitive and physical exercise with cognitive activity or physical exercise alone showed improvement in cognitive functions among older adults with MCI (Lam et al., 2015). However, nurse-led lifestyle risk factor modifications mentioned in the FINGER study such as diet and vascular risk monitoring were not included in this study.

Epidemiological studies show that reducing vascular risk factors has a beneficial role in reducing the incidence of dementia (Mangialasche et al., 2012; Ngandu et al., 2015; Olanrewaju et al., 2015; Vellas et al., 2014). Nurse-led interventions have recently been developed for diseases and specific conditions in Hong Kong, such as the risk assessment and management programme (RAMP) for diabetes and hypertension (Jiao et al., 2015; Yu et al., 2015). To our knowledge, no study has examined the effectiveness of either a nurse-led risk factor modification intervention or an intervention that combines nurse-led modification with mind-body physical exercise and cognitive training in the MCI population before. It can be a cost-effective way to examine the effectiveness of these two interventions together in Hong Kong Chinese which has a different culture and healthcare system. Our pilot study aimed to examine the feasibility and preliminary effectiveness of a multi-component intervention: cognitive training, mind-body physical exercise, and nurse-led risk factor modification (CPR) and a nurse-led risk factor modification (RFM) on reducing cognitive decline among the older adults aged between 60 and 80 years with MCI when compared to health advice (HA) in primary care. We hypothesized that CPR and RFM are feasible (having an acceptable recruitment rate of 25%, a dropout rate of 20%, and an adherence rate of 70%) and would have preliminary benefits on older adults with MCI.

## Material and Methods

### Study design

This was a pilot, 6-month open-labeled randomized controlled trial in primary care and community settings in Hong Kong from March to October 2018. This study included three experimental groups: integrated cognitive training and mind-body physical exercise plus nurse-led risk factor modification (CPR), nurse-led risk factor modification (RFM), and regular health advice (HA). Participants were randomly allocated to one of the three arms using computer-generated allocation sequencing with sequentially numbered, opaque sealed.

The medical and nurse assessments were conducted at a University affiliated research and training clinic. Cognitive training and physical exercise took place in the activity room at the research clinic. The study protocol was approved by the Joint Chinese University of Hong Kong - New Territories East Cluster Clinical Research Ethics Committee (CREC 2016.342), and the trial was registered on the Chinese Clinical Trial Registry (ChiCTR1800015324). The authors confirm that all ongoing and related trials for this intervention are registered.

### Participants

Participants were public primary care patients who voluntarily joined a community-based primary care programme including patients aged 60 or above with two or more chronic conditions from the New-Territories East Cluster of Hong Kong. All the participants were recruited in March 2018. Interested participants who met the following criteria were included in the study: (1) aged 60 to 80 years; (2) scored 19–21 after adjusting for years of educational (+1 point if <6 years) on the Montreal Cognitive Assessment Hong Kong version (HK-MoCA) (Yeung et al., 2014); and (3) being physically stable and without life-threatening diseases.

Subjects were excluded if they had any of the following (1) a diagnosis of dementia; (2) participating in any structured cognitive training, regular physical exercise including Tai Chi or other lifestyle modifications (e.g., diet) with no restrictions imposed on leisure activity; (3) history of bipolar affective disorder or psychosis; and (4) one or more significant communicative impairments. Any use of anti-dementia and other psychotropic medications was accepted but should be kept constant (with no change of dosage) for at least three months before enrollment and throughout the study period.

### Intervention

#### Risk factor modification group (RFM)

The components of RFM were modelled after the FINGER trial (Ngandu et al., 2015) with modifications based on local common public primary care practice. Participants were interviewed by the study clinician at baseline and month 6. Detailed medical history and physical examination including blood pressure, weight, and body mass index (BMI), hip and waist circumference were obtained. Participants in this group also met the nurse at baseline, 3, and 6 months to modify individual risk factors for dementia as suggested by established treatment protocols for cognitive impairment or dementia (Cooper et al., 2015a; Deckers et al., 2015; Li et al., 2016). In brief, RFM interventions were divided into two parts: nutritional intervention and management of metabolic and vascular risk factors. The nutritional intervention included tailor-made diet, discussions, and practical exercises to facilitate lifestyle changes, and energy restriction if needed. The diet goals were to achieve adequate consumption of fruit and vegetables (2 portions of fruits and 3–4 portions of vegetables each day); low-fat options in meat products; sucrose intake 50 g/day, and consumption of fish in at least two portions per week, which were the same as the intervention in the FINGER trial (Ngandu et al., 2015). Risk factors included suboptimal blood pressure or lipid or glucose control, high body mass index, smoking, physical inactivity, and an unhealthy diet. Both oral and written information on the importance of reducing these risk factors were given to the participants. An important part of the meeting with the nurse was motivating participants to make necessary lifestyle changes related to physical activity, dietary intake, smoking behavior, and medication use. Physical examinations including blood pressure, weight, and BMI, hip and waist circumference were also examined by the study physician. Motivational interviewing which used a guide toward change call FRAMES (feedback, responsibility, advice, menu, empathy, and self-efficacy) was applied to motivate the behavioral changes related to physical activity, dietary intake, smoking behavior and medication use which have been used locally (Bien, Miller & Tonigan, 1993; Thompson et al., 2011). Participants were recommended to contact their physician or clinic if necessary.

#### Combined mind-body physical exercise, cognitive training and risk factor modification group (CPR)

Same as the RFM group, participants in this group were interviewed by the study clinician at baseline and month 6. Detailed medical history and physical examination including blood pressure, weight, and BMI, hip, and waist circumference were obtained. Both cognitive and mind-body physical exercise followed established interventions previously used and evaluated for older adults with cognitive impairment (Lam et al., 2012). The risk factor modification component was the same as has been mentioned above in the RFM group. Participants received a 30-minute session of 24-forms simplified Tai Chi given by a Tai Chi master or trained physical therapists under the supervision of the team physician, which lasted for three times a week over 12 weeks (Lam et al., 2012). After 12 weeks of Tai Chi training, participants were advised to practice following a video or an audio recording in individual or group either in elderly centers or in their neighborhoods to maintain Tai Chi practices. For cognitive training, the main activity carried out in this pilot was Rummikub, which is an easy-to-learn and entertaining tile-based game. It is similar to mah-jong in China, where strategy is needed to win the game. One-hour group sessions were arranged three times a week for 12 weeks.

#### Heath Advice (HA) control

Participants in the HA group were interviewed by the study clinician at screening only for a detailed medical history. Physical examination including blood pressure, weight, and BMI, hip and waist circumference. Participants in the group also received health advice from a nurse in the form of booklets at baseline, which were from the ren.

#### Measurements

Demographic data, including age, gender, marital status, body mass index, waist-to-hip ratio, smoking, and alcohol drinking, common diseases were collected at baseline. Secondary outcomes were measured at baseline and 6 months post baseline. The assessors who rated the participants were blinded to the intervention arms. All the data were locked in a cabinet or saved in pass-word-protected computers to ensure data safety. No personal identity was revealed in any reports or publications. Only the research team had access to the data. The authors did not have access to information that could identify individual participants during or after data collection.

#### Primary outcome

The primary outcome was to assess the feasibility of the study because this is a pilot study of a larger clinical trial. The assessment included the recruitment rate, the dropout rate, and the adherence rates of mind-body physical exercise, cognitive training, and risk factor modification sessions, respectively. The recruitment rate was defined as the number of participants enrolled divided by the number invited. Dropout was defined as who did not finish the baseline or follow up assessment. The adherence rate was defined as the proportion of the intervention sessions completed. A Recruitment of more than 25%, a dropout rate of less than 20%, and an adherence rate of more than 70% were deemed acceptable (Cooper et al., 2015b; Thabane et al., 2010).

#### Secondary outcomes

The primary outcome to be tested in the future trial was tested as a secondary outcome in this pilot. The Chinese version of the Alzheimer’s Disease Assessment Scale-Cognitive Subscale (ADAS-Cog). ADAS-Cog is a frequently-used cognitive function assessment tool that has been well validated in the elderly Chinese population in Hong Kong (Chu et al., 2000). The total score of ADAS-Cog ranges from 0 to 70, with higher scores representing greater impairment (Chu et al., 2000).

*HK-MoCA.* HK-MoCA is a brief cognitive screening instrument for identifying MCI and dementia in Chinese older adults (Yeung et al., 2014). An extra point will be added if years of education is less than six. A total score of 19 to 21 represents MCI, and 18 or below suggests dementia.

*Clinical Dementia Rating (CDR) sum of box.* CDR is a global assessment measuring the influence of cognitive loss on the ability to conduct everyday activities, ranging from 0-3 (no dementia to severe dementia) (Morris, 1997).

*The Disability Assessment for Dementia (DAD).* The Chinese version of the DAD scale assesses functional disability activities of daily living, which have been subdivided into initiation, planning and organization, and effective performance (Mok et al., 2005). A total score ranges from 0 to 100, with higher scores representing less functional disabilities.

*Quality of life.* Quality of life was measured by the validated Chinese version of the EuroQoL 5-D Questionnaire, the five-level version (EQ-5D), and its visual analogue scale (EQ-VAS) (Luo et al., 2003).

*Depression and anxiety.* Depression and anxiety were measured by the Chinese versions of the Geriatric Depression Scale with a maximum score of 15 (GDS-15) (Chiu et al., 1994) and the Geriatric Anxiety Scale with a maximum score of 20 (GAS-20) (Lin et al., 2016), respectively.

*Physical activity.* The level of physical activity was quantified by using the Physical Activity Scale for the Elderly (PASE) (Vaughan & Miller, 2013), with total scores ranging from zero to 400 or more.

*Health service utilization.* The frequency of public and private health service utilization over the past six months and the costs of utilizing these services were recorded.

*Diet.* Dietary intake was measured using a shortened version of the food frequency questionnaire, which has been validated in Hong Kong (Yu et al., 2011).

### Data and statistical analysis

Primary outcomes were represented as percentages and 95% confidence interval (CI). Other continuous data were represented as mean and standard deviation (SD) for normal data, medians and quartiles for non-normal data. Categorical data were represented as counts and percentages. One sample proportion test was used to test the recruitment, adherence, and dropout rates compared to the acceptable rates. Kolmogorov–Smirnov testing was applied to test normality for continuous data. Analysis of variance (ANOVA) for normal data, Kruskal–Wallis test for non-normal data, or chi-square test for categorical data were used to compare the baseline variables of the three groups. The paired differences between pairs of the three intervention groups at baseline were assessed by post-hoc Least-Significance-Differnece (LSD) Methods. The within-group changes between pre-intervention and post-intervention were analyzed by paired *t*-test for normal data and Wilcoxon Signed Rank test for non-normal data. Sensitivity analysis was performed using full analysis set (FAS). Linear mixed models (LMM) for repeated measures were used to investigate the significant changes over time and between groups for secondary outcome measures. The fixed components of the models included a group × time interaction, which was adjusted for baseline data. Post-hoc LSD methods were performed among significant outcomes in LMM to assess the pairwise difference of the changes in three groups. LMM was conducted using STATA version 13, and all other statistical analyses were performed using SPSS version 23.0. Significance level for all tests was set at a *P* value less than 0.05 (two-tailed).

## Results

**Figure 1:**
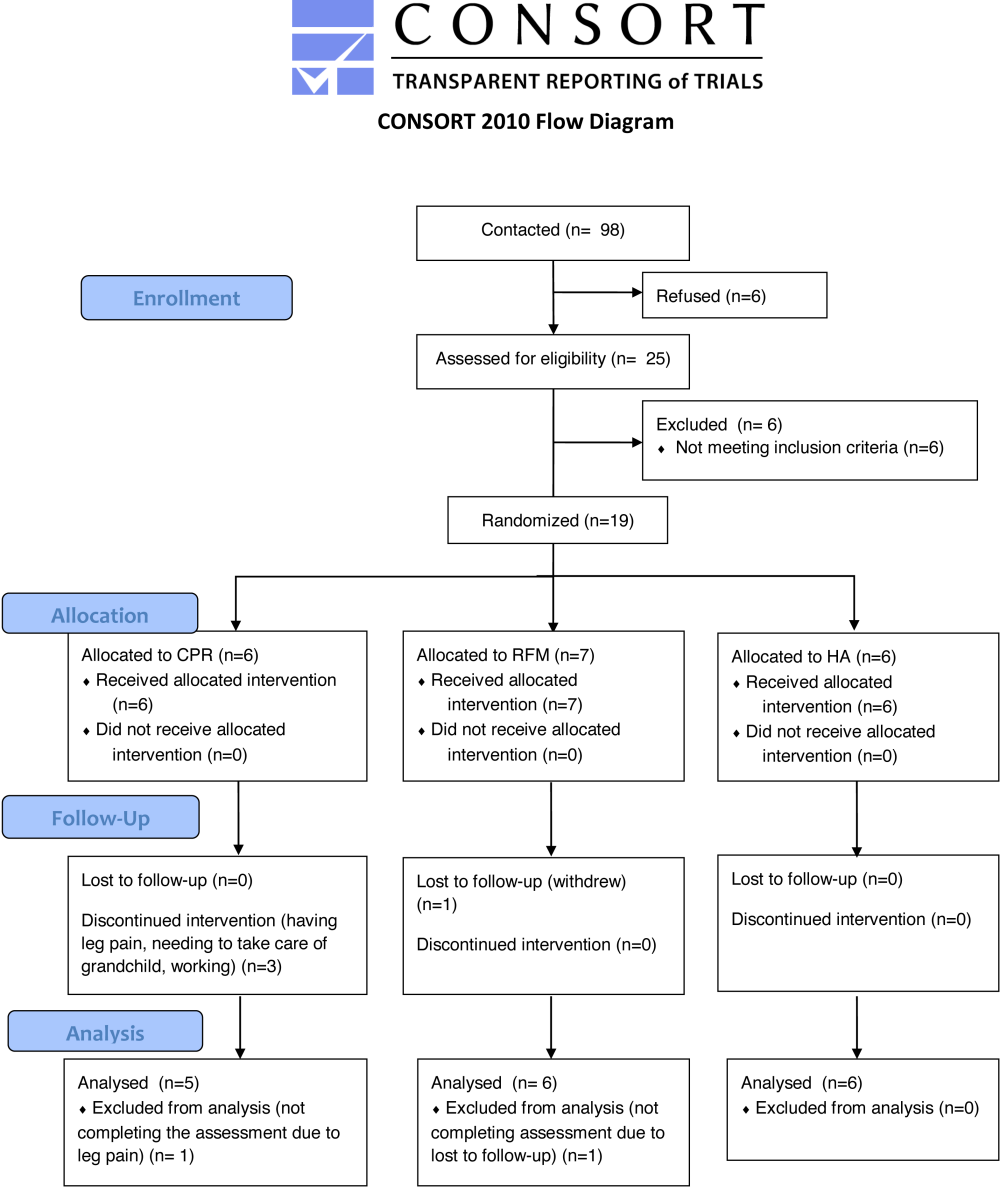
Flowchart for study participants. CPR, cognitive training, mind-body physical exercise, and nurse-led risk factor modification; HA, health advice; RFM, nurse-led risk factor modification.

A total of 98 participants were contacted and 25 were recruited and screened, among whom 6 participants were excluded for not meeting the eligibility criteria. Nineteen participants (19%, 95% CI [12–29]%, *P* = 0.243) were finally randomized, with 6 assigned to CPR, 7 assigned to RFM, and 6 assigned to HA. One participant in the RFM group withdrew from the study. Among the 36 sessions in the CPR group, 53% (95% CI [36–70]%, *P* = 0.038) of the Tai Chi exercise sessions and 54% (95% CI [37–71]%, *P* = 0.051) of cognitive sessions were completed respectively. The main reasons that participants could not attend the sessions included having leg pain, needing to take care of grandchild, working, and traveling. The participants in the CPR and RFM group came back to see the nurse at baseline, 3 months and 6 months, with the adherence rate of 89% (95% CI [65–98]%, *P* = 0.139) and 86% (95% CI [63–96]%, *P* = 0.182), respectively. Seventeen participants (5 in CPR, 6 in RFM, 6 in HA) completed the follow-up assessments at 6 months, with an overall dropout rate of 11% (95% CI [2–34]%, *P* = 0.456) among MCI patients. Participants completing both baseline and follow-up assessments were included in the data analysis (Fig. 1).

Table 1 shows the demographics, cognitive characteristics, quality of life, lifestyle (physical activities and diet), depression and anxiety, healthcare utilization, and common diseases of the participants at baseline. There were no significant differences between the three groups at baseline except the number of servings of bread, pasta, and rice eaten per day (*P* = 0.040). Post-hoc LSD testing showed that the number of servings of bread, pasta, and rice eaten per day were significantly different between CPR group and HA group (*P* = 0.019), and between RFM and HA group (*P* = 0.010).

**Table 1:** Baseline characteristics.

	Total (*n* = 19)	CPR (*n* = 6)	RFM (*n* = 7)	HA (*n* = 6)	*P*
Age, y	74.00 ± 5.24	70.67 ± 4.23	76.43 ± 4.47	74.50 ± 5.93	0.135
Male, n	5 (26.3)	2 (33.3)	2 (28.6)	1 (16.7)	1.000
BMI, kg/m^2^	25.70 ± 5.13	25.30 ± 4.47	24.69 ± 3.93	27.25 ± 7.19	0.678
WHR, %	91.33 ± 4.07	92.17 ± 4.58	92.06 ± 2.66	89.65 ± 5.03	0.497
Medical history, n	3 (2, 4)	2.5 (2, 3.25)	3 (2, 5)	3 (1.75, 3.25)	0.697
Smoker, n	2 (10.5)	0 (0)	1 (14.2)	1 (16.7)	1.000
Drinker, n	3 (15.8)	0 (0)	2 (28.6)	1 (16.7)	0.740
Servings of food eaten per day, n					
Bread/pasta/rice	5.67 ± 2.42	6.54 ± 1.90	6.77 ± 2.02	3.52 ± 2.09	0.040^*^
Vegetables	5.10 ± 2.49	5.13 ± 2.03	5.46 ± 2.68	4.65 ± 3.03	0.770
Fruits	2.17 ± 1.46	1.59 ± 1.21	2.39 ± 1.82	2.50 ± 1.27	0.345
Milk	0.07 (0.00, 1.00)	0.10 (0.00, 1.57)	0.86 (0.00, 1.00)	0.00 (0.00, 0.51)	0.561
Eggs	0.43 (0.21, 0.43)	0.36 (0.19, 0.58)	0.29 (0.21, 0.43)	0.43 (0.12, 1.03)	0.618
Meat	2.03 ± 1.55	1.87 ± 1.58	1.74 ± 1.49	2.53 ± 1.73	0.731
Fish and seafood	1.84 (0.62, 3.00)	1.35 (0.32, 3.64)	1.49 (0.60, 4.28)	2.19 (1.23, 3.39)	0.617
Soya	1.22 ± 1.95	0.63 ± 0.38	1.91 ± 2.99	1.02 ± 1.28	0.710
Oil	1.88 ± 1.65	1.83 ± 1.60	2.42 ± 1.48	1.31 ± 1.93	0.293
Sugar	0.35 (0.00, 1.00)	0.46 (0.10, 1.20)	0.14 (0.00, 0.64)	0.75 (0.11, 1.10)	0.535
Public health service utilization, n	5.05 ± 3.50	6.33 ± 5.428	4.86 ± 2.80	4.00 ± 1.41	0.532
Private health service utilization, n	2 (1, 4)	2.5 (0.75, 9)	3 (2, 4)	1.5 (0.75, 6.5)	0.918
Cost of health service utilization, HKD	500 (0, 2000)	2500 (450, 7000)	300 (0, 2000)	150 (0, 1175)	0.101
HK-MoCA	19 (19, 21)	20 (19, 21)	19 (19, 21)	19 (19, 20.25)	0.699
CDR sum of box	0.5 ± 0.0	0.5 ± 0.0	0.5 ± 0.0	0.5 ± 0.0	–
ADAS-Cog	13.08 ± 3.56	12.44 ± 3.25	13.40 ± 4.72	13.33 ± 2.76	0.882
Delay recall	2 (0, 4)	0 (0, 4.25)	3 (2, 5)	1 (0, 3)	0.243
DAD	94.06 ± 3.86	91.84 ± 3.13	94.53 ± 4.40	95.74 ± 3.30	0.206
EQ-5D	−0.32 (−0.48, −0.16)	−0.24 (−0.48, −0.10)	−0.48 (−0.48, −0.32)	−0.28 (−0.48, −0.04)	0.210
EQ-VAS	67.95 ± 22.78	62.67 ± 34.56	74.29 ± 16.18	65.83 ± 16.25	0.653
GDS-15	5 (1, 8)	7.5 (1.75, 9.25)	4 (1, 7)	4 (0.75, 6.75)	0.450
GAS-20	6 (1, 11)	10.5 (4.5, 17.25)	4 (0, 9)	2.5 (0.75, 8.5)	0.263
PASE	99.64 ± 36.65	110.36 ± 31.27	104.51 ± 42.71	83.25 ± 34.27	0.423

**Notes.**

ADAS-Cog the Alzheimer’s Disease Assessment Scale-Cognitive Subscale BMI body mass index CDR Clinical Dementia Rating CPR cognitive training, mind-body physical exercise, and nurse-led risk factor modification CSSA Comprehensive Social Security Assistance DAD the Disability Assessment for Dementia EQ-5D EuroQoL Questionnaire (quality of life, five-level version) EQ-VAS EuroQoL Questionnaire (quality of life, visual analogue scale) GAS-20 Geriatric Anxiety Scale GDS-15 Geriatric Depression Scale HA health advice HKD Hong Kong dollar HK-MoCA Montreal Cognitive Assessment Hong Kong version PASE Physical Activity Scale for the Elderly RFM nurse-led risk factor modification WHR waist-to-hip ratio

Quantitative data were presented as mean ± SD for normal data, and medians and quartiles for non-normal data. Qualitative data were represented as n (%). ANOVA was used for normal data, Kruskal-Wallis test for non-normal data, and chi-square test for qualitative data. * *P* < 0.05.

* *P* < 0.05.

Table 2 summarizes the differences in the secondary outcomes between baseline and follow up, and differences between groups at six months. Among outcomes related to cognitive function, the linear mixed model was conducted and there were group × time interactions on HK-MoCA favoring the RFM group (*P* < 0.001), DAD score favoring CPR group (*P* = 0.027), GAS-20 favoring CPR group (*P* = 0.026), number of servings of fish and seafood (*P* = 0.004), and sugar (*P* < 0.001) ate per day (Table 2). The results for post-hoc LSD testing were presented in Table S1. Within-group improvements were noted for all the three groups in HK-MoCA, but only the improvement in the RFM group was significant (4.50  ± 2.59, *P* = 0.008). The CPR group showed a significant reduction in the cost of health service utilization (−4000, quartiles: −6800 to −200, *P* = 0.043). At six months, the number of servings of fish and seafood ate per day decreased significantly (−1.10  ± 1.02, *P* = 0.047), and the number of servings of sugar ate per day increased significantly (2.69  ± 1.80, *P* = 0.015) in HA group. The results of FAS were presented in Table S2.

**Table 2:** Outcome comparison in CPR, RFM and HA Groups.

Outcomes	CPR (*n* = 5)			RFM (*n* = 6)			HA (*n* = 6)			*P*^b^
	Change	*P*^a^		Change	*P*^a^		Change	*P*^a^		
HK-MoCA	2.75 ± 4.03	0.266		4.50 ± 2.59	0.008^*^		2.33 ± 2.66	0.084		<0.001^*^
CDR sum of box	0.0 ± 0.0	–		0.0 ± 0.0	–		0.0 ± 0.0	–		
ADAS-Cog	−0.13 ± 6.45	0.967		−0.63 ± 3.37	0.666		0.84 ± 3.12	0.539		0.733
Delay recall	0.00 (0.00, 2.50)	0.180		−1.00 (−4.50, 0.00)	0.109		0.00 (−1.25, 2.00)	0.705		0.365
DAD	5.53 ± 5.75	0.098		2.83 ± 6.26	0.318		1.42 ± 5.49	0.555		0.027^*^
EQ-5D	0.01 (−0.03, 0.12)	0.465		0.13 (−0.04, 0.22)	0.138		−0.04 (−0.17, 0.14)	0.715		0.325
EQ-VAS	10.0 (−2.5, 27.0)	0.216		−5.0 (−12.5, 17.5)	0.891		2.5 (−15.0, 15.0)	0.892		0.658
GDS-15	0.00 (−1.00, 2.00)	0.564		1.00 (−1.50, 3.25)	0.715		0.00 (−2.00, 4.75)	0.461		0.301
GAS-20	−7.00 (−8.50, −3.00)	0.068		−1.50 (−7.50, 0.00)	0.068		−0.50 (−5.50, 2.50)	0.500		0.026^*^
PASE	8.44 ± 17.56	0.343		−4.99 ± 48.06	0.809		3.02 ± 37.57	0.851		0.836
Public health service utilization	1.00 (−2.50, 10.0)	0.786		2.50 (−0.75, 4.00)	0.248		−0.50 (−3.25, 3.75)	1.000		0.700
Private health service utilization	−2.0 (−13.5, 0.0)	0.109		−0.50 (−1.50, 4.25)	0.785		−0.50 (−3.00, 1.25)	0.496		0.592
Cost of health service utilization	−4000 (−6800, −200)	0.043^*^		0 (−650, 1625)	0.854		0 (-500, 3650)	0.285		0.570
Servings of food eat per day										
Bread/pasta/rice	−1.38 ± 2.82	0.334		−0.54 ± 2.77	0.652		1.22 ± 2.65	0.313		0.660
Vegetables	−1.06 ± 2.46	0.390		−0.70 ± 1.41	0.278		0.65 ± 4.25	0.722		0.826
Fruits	0.28 ± 0.58	0.342		−0.84 ± 1.76	0.297		0.06 ± 1.92	0.947		0.710
Milk	−0.23 ± 0.68	0.496		0.25 ± 0.65	0.388		0.22 ± 0.49	0.291		0.141
Eggs	0.47 ± 0.95	0.334		0.10 ± 0.38	0.564		0.48 ± 0.64	0.127		0.053
Meat	−0.09 ± 1.59	0.909		−0.23 ± 1.02	0.604		−0.50 ± 3.13	0.710		0.497
Fish and seafood	−1.12 ± 1.94	0.268		−0.63 ± 1.47	0.343		−1.10 ± 1.02	0.047^*^		0.004^*^
Soya	−0.06 ± 0.75	0.860		−1.70 ± 3.41	0.277		−0.49 ± 1.42	0.437		0.053
Oil	1.69 ± 2.72	0.239		0.17 ± 1.89	0.837		1.65 ± 2.20	0.125		0.054
Sugar	0.14 ± 1.54	0.845		0.94 ± 0.99	0.068		2.69 ± 1.80	0.015^*^		<0.001^*^

**Notes.**

ADAS-Cog the Alzheimer’s Disease Assessment Scale-Cognitive Subscale CDR Clinical Dementia Rating CPR cognitive training, mind-body physical exercise, and nurse-led risk factor modification DAD the Disability Assessment for Dementia EQ-5D EuroQoL Questionnaire (quality of life, five-level version) EQ-VAS EuroQoL Questionnaire (quality of life, visual analogue scale) GAS-20 Geriatric Anxiety Scale GDS-15 Geriatric Depression Scale HA health advice HK-MoCA Montreal Cognitive Assessment Hong Kong version PASE Physical Activity Scale for the Elderly RFM nurse-led risk factor modification

Normal data were presented as mean ± SD, and non-normal data were presented as medians and quartiles.

a Difference between baseline and follow up. Paired *t*-test was used for normal data, Wilcoxon Signed Rank test for non-normal data.

b Difference between groups using linear mixed model (group × time interaction adjusted for baseline data) ( *n* = 19).

* *P* < 0.05.

## Discussion

This study explored the feasibility and potential health benefits of RFM and a multi-domain intervention CPR when compared with HA on cognitive function in older adults with MCI. The main focus of this study was on evaluating the overall feasibility of the trial process, including the ability to recruit participants, increase adherence to the intervention sessions, decrease dropout from the study, and the ability to assess the preliminary effects of the interventions. The result of this pilot study can guide the future large-scale trial.

This study showed that it was feasible to conduct the multi-component intervention on public primary care patients with MCI. The recruitment rate was 19% in this pilot, which was lower than the preliminary goal of 25%. Nevertheless, the process of recruitment was different from the future large-scale trial. In this study, for convenience, participants were public primary care patients who voluntarily joined another community-based primary care programme in Hong Kong. Participating in a new project may cause burdens to the participants when they have complied with another programme before. In the future study, participants will be recruited in the community and primary care settings through geriatric day centers and clinics, and elderly social centers which provide services for the elderly. Participants will also be recruited from the public general outpatient clinics and send out invitation letters and posters to community councils for facilitating subject recruitment. All interested participants will undergo screening for MCI.

High adherence showed in risk factor modification intervention, which was up to more than 85% in both CPR and RFM group. Participants came back to see the nurses for about 30 min with an interval of three months. It did not require too much time from the participants. The adherence rates of the training sessions were low but acceptable. In previous non-pharmacological interventions among the MCI population in Hong Kong, the adherence rates were around 53.8% to 79.0% (Lam et al., 2015; Lam et al., 2012). Family issues and poor health conditions may be important barriers for older adults to participate in the training sessions in this pilot, including having leg pain, needing to take care of grandchild, working, and travelling. In this study, with reminders from the study coordinating staff, very few people failed to participate in the intervention because of forgetting. Phone reminder has been shown to support adherence in the MCI population because this population may need to rely on reminder due to memory impairment (Van der Wardt et al., 2017). Strategies to ensure adherence of Tai Chi exercise and cognitive activities in the future trial will be applied, such as discussions with the physical trainer of future exercise time and location and possible group exercise, and also discussion with a nurse of preferences, possible barriers, and facilitators in doing the exercise and cognitive activities during the clinical visits or assessments if the adherence is suboptimal. A refreshing course may also be provided once a month after the class.

Preliminary improvements were observed in HK-MoCA, GAS-20, cost of health service utilization, and DAD. This showed CPR and RFM may have preliminary beneficial effects on MCI. The effects will be tested and verified in the future study. It is observed that the intake of many kinds of food decreased in both CPR and RFM group. At baseline, most participants were overweight or obese. Risk factor modification provided tailor-made dietary suggestions and helped participants to reduce excessive food intake. Participants still met the recommended amount of food after the decrease in consumption. However, in the HA group, who had the highest BMI, their diet became worse, due to the increased food consumption, a significant decrease in fish and seafood, and a significant increase in sugar intake.

### Limitations

The study has several limitations. First, because it is a pilot study with a small sample size with a follow-up time of six months, the results should be interpreted as preliminary. The results may have low statistical power and may not be representative, although they were largely consistent with our hypothesis. Many potential effects may also be prohibited from emerging due to the sample size. The current results on effectiveness were for preliminary references only. A larger RCT with longer intervention and long-term follow-up will be conducted to confirm these findings. Second, the level of adherence to the mind-body exercise programme and cognitive training might have resulted in the attenuation of the potential effects of the interventions. Strategies are needed in a future study to improve training adherence. Third, Servings of food ate per day were measured in this study to investigate the impact of specific food types but may lead to bias if there is no fixed criterion to evaluate it. Total energy and nutrients intake, as well as the concept of the dietary index, can be used to evaluate the overall dietary quality in our future research (Kwan et al., 2013).

## Conclusions

In summary, the results of this study preliminarily show that a multi-domain approach CPR including physical exercise, cognitive training, and nurse-led risk factor modification is feasible and may have preliminary beneficiary effects on reducing the cognitive decline of elderly patients with MCI in the primary care setting. It is feasible to eventually perform the larger scaled randomized controlled study, which is planned to further examine the effects of the multi-domain approach in primary care.

##  Supplemental Information

10.7717/peerj.9845/supp-1 Supplemental Information 1 Post-hoc Least-Significance-Difference (LSD) analysis among outcomes significant in linear mixed model CPR: cognitive training, mind-body physical exercise, and nurse-led risk factor modification; DAD: The Disability Assessment for Dementia; GAS-20: Geriatric Anxiety Scale; HA: health advice; HK-MoCA: Montreal Cognitive Assessment Hong Kong version; RFM: nurse-led risk factor modification. * *P* < 0.05

10.7717/peerj.9845/supp-2 Supplemental Information 2 Outcome Comparison in CPR, RFM and HA Groups using full analysis set (FAS) analysis ADAS-Cog: the Alzheimer’s Disease Assessment Scale - cognitive subscale; CDR: Clinical Dementia Rating; CPR: cognitive training, mind-body physical exercise, and nurse-led risk factor modification; DAD: The Disability Assessment for Dementia; EQ-5D: EuroQoL Questionnaire (quality of life, five-level version); EQ-VAS: EuroQoL Questionnaire (quality of life, visual analogue scale); GAS-20: Geriatric Anxiety Scale; GDS-15: Geriatric Depression Scale; HA: health advice; HK-MoCA: Montreal Cognitive Assessment Hong Kong version; PASE: Physical Activity Scale for the Elderly; RFM: nurse-led risk factor modification. Normal data were presented as mean±SD, and non-normal data were presented as medians and quartiles. † Difference between baseline and follow up. Paired t-test was used for normal data, Wilcoxon Signed Rank test for non-normal data. * *P* < 0.05

10.7717/peerj.9845/supp-3 Supplemental Information 3 Dataset for the assessment at all time points

10.7717/peerj.9845/supp-4 Supplemental Information 4 CONSORT Checklist

10.7717/peerj.9845/supp-5 Supplemental Information 5 Trial protocol

## Additional Information and Declarations

### Data Availability

The following information was supplied regarding data availability: Data are available in the Supplemental Files.

### Clinical Trial Registration

The following information was supplied regarding Clinical Trial registration: The trial was registered on the Chinese Clinical Trial Registry (ChiCTR1800015324).
