# Supplementary material for: A pilot feasibility randomized controlled trial on combining mind-body physical exercise, cognitive training, and nurse-led risk factor modification to reduce cognitive decline among older adults with mild cognitive impairment in primary care
Source: PeerJ. 2020 Sep 7;8:e9845. doi: 10.7717/peerj.9845 (PMC7482623; doi:10.7717/peerj.9845)
Supplement: Supplemental Information 1 — CPR: cognitive training, mind-body physical exercise, and nurse-led risk factor modification; DAD: The Disability Assessment for Dementia; GAS-20: Geriatric Anxiety Scale; HA: health advice; HK-MoCA: Montreal Cognitive Assessment Hong Kong version; RFM: nurse-led risk factor modification. * P < 0.05 [file peerj-08-9845-s001.docx]

**Supplemental Table S1. Post-hoc Least-Significance-Difference (LSD) analysis among outcomes significant in linear mixed model**

| Outcomes | CPR vs RFM | | |  | CPR vs HA | | |  | RFM vs HA | | |
| --- | --- | --- | --- | --- | --- | --- | --- | --- | --- | --- | --- |
|  | CPR (n=5) | RFM (n=6) | *P* |  | CPR (n=5) | HA (n=6) | *P* |  | RFM (n=6) | HA (n=6) | *P* |
| HK-MoCA | 2.75±4.03 | 4.50±2.59 | 0.222 |  | 2.75±4.03 | 2.33±2.66 | 0.942 |  | 4.50±2.59 | 2.33±2.66 | 0.227 |
| DAD | 5.53±5.75 | 2.83±6.26 | 0.459 |  | 5.53±5.75 | 1.42±5.49 | 0.265 |  | 2.83±6.26 | 1.42±5.49 | 0.681 |
| GAS-20 | -6.00±3.54 | -3.17±3.86 | 0.249 |  | -6.00±3.54 | -1.17±4.17 | 0.049* |  | -3.17±3.86 | -1.17±4.17 | 0.388 |
| Fish and seafood | -1.12±1.94 | -0.63±1.47 | 0.595 |  | -1.12±1.94 | -1.10±1.02 | 0.981 |  | -0.63±1.47 | -1.10±1.02 | 0.594 |
| Sugar | 0.14±1.54 | 0.94±0.99 | 0.387 |  | 0.14±1.54 | 2.69±1.80 | 0.013* |  | 0.94±0.99 | 2.69±1.80 | 0.061 |

CPR: cognitive training, mind-body physical exercise, and nurse-led risk factor modification; DAD: The Disability Assessment for Dementia; GAS-20: Geriatric Anxiety Scale; HA: health advice; HK-MoCA: Montreal Cognitive Assessment Hong Kong version; RFM: nurse-led risk factor modification.

* *P*<0.05
