# Supplementary material for: A pilot feasibility randomized controlled trial on combining mind-body physical exercise, cognitive training, and nurse-led risk factor modification to reduce cognitive decline among older adults with mild cognitive impairment in primary care
Source: PeerJ. 2020 Sep 7;8:e9845. doi: 10.7717/peerj.9845 (PMC7482623; doi:10.7717/peerj.9845)
Supplement: Supplemental Information 2 — ADAS-Cog: the Alzheimer’s Disease Assessment Scale - cognitive subscale; CDR: Clinical Dementia Rating; CPR: cognitive training, mind-body physical exercise, and nurse-led risk factor modification; DAD: The Disability Assessment for Dementia; EQ-5D: EuroQoL Questionnaire (quality of life, five-level version); EQ-VAS: EuroQoL Questionnaire (quality of life, visual analogue scale); GAS-20: Geriatric Anxiety Scale; GDS-15: Geriatric Depression Scale; HA: health advice; HK-MoCA: Montreal Cognitive Assessment Hong Kong version; PASE: Physical Activity Scale for the Elderly; RFM: nurse-led risk factor modification. Normal data were presented as mean±SD, and non-normal data were presented as medians and quartiles. † Difference between baseline and follow up. Paired t-test was used for normal data, Wilcoxon Signed Rank test for non-normal data. * P < 0.05 [file peerj-08-9845-s002.docx]

**Supplemental Table S2. Outcome Comparison in CPR, RFM and HA Groups using full analysis set (FAS) analysis**

| Outcomes | CPR (n=6) | |  | RFM (n=7) | |  | HA (n=6) | |  |
| --- | --- | --- | --- | --- | --- | --- | --- | --- | --- |
|  | Change | *P*† |  | Change | *P*† |  | Change | *P* † |  |
| HK-MoCA | 1.83±3.43 | 0.247 |  | 3.85±2.91 | 0.013* |  | 2.33±2.66 | 0.084 |  |
| CDR sum of box | 0.0±0.0 | - |  | 0.0±0.0 | - |  | 0.0±0.0 | - |  |
| ADAS-Cog | -0.11±5.77 | 0.966 |  | -0.54±3.09 | 0.660 |  | 0.84±3.12 | 0.539 |  |
| Delay recall | 0.00 (0.00, 2.50) | 0.180 |  | 0.00 (-4.00, 0.00) | 0.109 |  | 0.00 (-1.25, 2.00) | 0.705 |  |
| DAD | 4.61±5.62 | 0.101 |  | 2.43±5.82 | 0.311 |  | 1.42±5.49 | 0.555 |  |
| EQ-5D | 0.01 (-0.03, 0.12) | 0.465 |  | 0.10 (0.00, 0.19) | 0.138 |  | -0.04 (-0.17, 0.14) | 0.715 |  |
| EQ-VAS | 10.0 (-2.5, 27.0) | 0.216 |  | 0 (-10.0, 10.0) | 0.891 |  | 2.5 (-15.0, 15.0) | 0.892 |  |
| GDS-15 | 0.00 (-1.00, 2.00) | 0.564 |  | 0.00 (0.00, 3.00) | 0.715 |  | 0.00 (-2.00, 4.75) | 0.461 |  |
| GAS-20 | -7.00 (-8.50, -3.00) | 0.068 |  | -1.00 (-7.00, 0.00) | 0.068 |  | -0.50 (-5.50, 2.50) | 0.500 |  |
| PASE | 7.04±16.08 | 0.333 |  | -4.28±43.92 | 0.805 |  | 3.02±37.57 | 0.851 |  |
| Public health service utilization | 1.00 (-2.50, 10.0) | 0.786 |  | 2.00 (0.00, 3.00) | 0.248 |  | -0.50 (-3.25, 3.75) | 1.000 |  |
| Private health service utilization | -2.0 (-13.5, 0.0) | 0.109 |  | 0.00 (-1.00, 3.00) | 0.785 |  | -0.50 (-3.00, 1.25) | 0.496 |  |
| Cost of health service utilization | -4000 (-6800, -200) | 0.043* |  | 0 (-200, 1500) | 0.854 |  | 0 (-500, 3650) | 0.285 |  |
| Servings of food eat per day |  |  |  |  |  |  |  |  |  |
| Bread/pasta/rice | -1.15±2.58 | 0.324 |  | -0.46±2.53 | 0.645 |  | 1.22±2.65 | 0.313 |  |
| Vegetables | -0.88±2.24 | 0.379 |  | -0.60±1.31 | 0.272 |  | 0.65±4.25 | 0.722 |  |
| Fruits | 0.23±0.53 | 0.322 |  | -0.72±1.64 | 0.291 |  | 0.06±1.92 | 0.947 |  |
| Milk | -0.19±0.62 | 0.485 |  | 0.21±0.60 | 0.380 |  | 0.22±0.49 | 0.291 |  |
| Eggs | 0.39±0.87 | 0.324 |  | 0.08±0.35 | 0.556 |  | 0.48±0.64 | 0.127 |  |
| Meat | -0.07±1.42 | 0.906 |  | -0.20±0.94 | 0.597 |  | -0.50±3.13 | 0.710 |  |
| Fish and seafood | -0.93±1.80 | 0.260 |  | -0.54±1.36 | 0.336 |  | -1.10±1.02 | 0.047* |  |
| Soya | -0.05±0.67 | 0.856 |  | -1.46±3.18 | 0.271 |  | -0.49±1.42 | 0.437 |  |
| Oil | 1.40±2.53 | 0.232 |  | 0.14±1.73 | 0.834 |  | 1.65±2.20 | 0.125 |  |
| Sugar | 0.13±1.38 | 0.840 |  | 0.81±0.98 | 0.071 |  | 2.69±1.80 | 0.015* |  |

ADAS-Cog: the Alzheimer’s Disease Assessment Scale - cognitive subscale; CDR: Clinical Dementia Rating; CPR: cognitive training, mind-body physical exercise, and nurse-led risk factor modification; DAD: The Disability Assessment for Dementia; EQ-5D: EuroQoL Questionnaire (quality of life, five-level version); EQ-VAS: EuroQoL Questionnaire (quality of life, visual analogue scale); GAS-20: Geriatric Anxiety Scale; GDS-15: Geriatric Depression Scale; HA: health advice; HK-MoCA: Montreal Cognitive Assessment Hong Kong version; PASE: Physical Activity Scale for the Elderly; RFM: nurse-led risk factor modification.

Normal data were presented as mean±SD, and non-normal data were presented as medians and quartiles.

† Difference between baseline and follow up. Paired t-test was used for normal data, Wilcoxon Signed Rank test for non-normal data.

* *P*<0.05
