# Supplementary material for: A pilot feasibility randomized controlled trial on combining mind-body physical exercise, cognitive training, and nurse-led risk factor modification to reduce cognitive decline among older adults with mild cognitive impairment in primary care
Source: PeerJ. 2020 Sep 7;8:e9845. doi: 10.7717/peerj.9845 (PMC7482623; doi:10.7717/peerj.9845)
Supplement: Supplemental Information 5 [file peerj-08-9845-s005.pdf]

PROPOSED RESEARCH PROJECT

a) Title:

Combining mind body physical exercise, cognitive training, and nurse-led risk factor modification to enhance cognition among older adults with mild cognitive impairment: a randomized controlled trial

b) Introduction:

Dementia and cognitive impairments have become important public health problems due to population ageing and changes in lifestyles and health profiles.[1-3] It has been estimated by the World Health Organization (WHO) that the total number of new cases of dementia each year worldwide was nearly 7.7 million in 2010 and that the number thereof is expected to double every 20 years.[1] Systematic reviews show that the age-standardized prevalence of dementia has been increasing rapidly and that it varies from 5 to 8%.[4] In Hong Kong, the latest study has shown that over 10% of older adults aged 70 or above suffer from dementia, with the predominant diagnosis being of Alzheimer's disease.[5]

The WHO has identified that the key strategies for tackling dementia include early diagnosis and prevention, as well as optimizing the physical health, cognition, activity, and well-being of sufferers.[1] Among all risk factors for dementia, hypertension, elevated cholesterol, obesity, diabetes, physical inactivity, smoking, and depression have been identified as the most important modifiable ones.[6-11] In Hong Kong, a recent cohort conducted by our investigators on 15,589 community-dwelling elderly confirmed these findings and showed that vascular and lifestyle risk factors, such as hypertension, diabetes mellitus, depression, and physical inactivity, were associated with incident dementia over a six-year period.[12] Moreover, physical exercises, such as aerobic and mind-body exercises (*e.g.*, Tai Chi), were associated with reduced risk.[12]

Epidemiological studies have demonstrated the association of risk factors and dementia, and therefore interventional studies are now needed to examine whether addressing these risk factors can prevent the occurrence or progression of the cognitive impairment associated with dementia. To address the various risk factors simultaneously, multi-component (complex) interventions have been advocated as the most appropriate public health strategies for the prevention of cognitive decline.[13-15] The Finnish Geriatric Intervention Study to Prevent Cognitive Impairment and Disability (FINGER) is a recent large randomized controlled trial conducted to evaluate the effectiveness of a two-year multi-domain intervention, including lifestyle, cognitive training, and vascular risk monitoring, to reduce cognitive decline among the elderly aged 60–77 years over a period of 2 years.[16] It showed that the multi-domain intervention could reduce or maintain cognitive functioning among the elderly at risk of dementia. However, randomized controlled trials that evaluated the effectiveness of multi-domain intervention or nurse-led risk factor modification for preventing cognitive decline among the elderly are rare although epidemiological studies support the beneficial effects of vascular risk factor reduction in the reduction of dementia incidence.[14,16-18]

In Hong Kong, Lam *et al*. (2015),[19] of our investigator team have demonstrated that combined physical activity and cognitive training, when compared to either type of intervention alone, have superior outcomes in reducing cognitive and memory decline among the elderly with mild cognitive impairments (MCI). They also have demonstrated that it is feasible and practical to recruit elderly participants with MCI in the community to participate in combined physical activity and cognitive training with high compliance over a one-year period.[19] However, unlike the FINGER trial, nurse-led lifestyle and health risk modification intervention was not included as part of the complex intervention and whether nurse-led structured lifestyle and risk factor modification was effective or whether it can confer additional benefits remains unknown. With the recent development of employing nurse-led

interventions for diseases and specific conditions in Hong Kong, *e.g.*, the risk assessment and management programme (RAMP) for diabetes and hypertension,[20,21] examining the effectiveness of a nurse-led intervention alone or in combination with other interventions for the prevention of dementia among the elderly with cognitive impairment will provide timely evidence that is implementable in primary care for policy-makers and researchers to plan for preventive strategies to reduce the incidence of dementia.[13,22,23] Well-designed randomized controlled trials conducted in primary care and community settings are therefore urgently needed to inform local practice and health policy regarding managing the epidemic of cognitive impairment and dementia.

The current proposed study aims to identify older adults with MCI in primary care and community settings to evaluate the effectiveness of a complex intervention consisting of nurse-led risk factor modification, mind body physical activity, and cognitive training or a nurse-led risk factor modification when compared to a health advice control to reduce cognitive decline. Since no studies have been conducted to evaluate the effectiveness of either a nurse-led risk factor modification intervention or an intervention that combines nurse-led modification with mind body physical activity and cognitive training before, conducting a multi-arm intervention study can be a cost effective way to evaluate the effectiveness of these two interventions simultaneously.

We propose to study only those elderly with MCI to reduce the sample size required; this is because the number needed to examine the effects of a multi-domain intervention among the at-risk elderly (who have not already developed cognitive impairments) would be very large. Moreover, targeting the elderly who have MCI presents a ‘golden window’ for dementia prevention and is a more cost effective and feasible strategy within the time frame and the budget supported by the current grant.

c) Aims and Hypotheses to be Tested:

This is a pragmatic trial with intervention and control components designed based on feasibility and implementability in primary care settings. The aim of this study is to examine the effectiveness of a complex intervention: cognitive training, mind body physical exercise, and nurse-led risk factor modification (CPR) on reducing cognitive decline among the older adults aged between 60 and 80 years with MCI when compared to health advice (HA) in primary care.

The secondary objective is to examine the effectiveness of a nurse-led risk factor modification (RFM) among elderly aged 60-80 years with MCI when compared to health advice (HA) in the community. Since studies have already been conducted by our co-investigators on the effectiveness of cognitive training and physical exercise on the reduction of cognitive decline among elderly with MCI, this group is not included in our current study to reduce cost.

Hypotheses:

Primary hypothesis:

1. We hypothesize that CPR would be superior to HA in reducing cognitive decline among older adults with MCI in primary care.

Secondary Hypotheses:

2. We hypothesize that RFM would be superior to HA in reducing cognitive decline among older adults with MCI but that CPR would have larger effects in reducing cognitive decline among older adults with MCI when compared to RFM in primary care

d) Plan of Investigation:

(i) Subjects

Four hundred and twenty three older adults aged between 60 and 80 years with MCI in primary care and community settings in Hong Kong.

(ii) Methods

This will be a randomized controlled trial with elderly participants allocated to one of the three arms (regular health advice (HA), nurse-led risk factor modification (RFM) and integrated cognitive training and mind body physical exercise plus nurse-led risk factor modification (CPR)) with a 1:1:1 ratio over 12 months (each with 135 participants). This trial is designed to model after real life primary care practice and takes into account the pragmatic implications for future implementation of intervention components (translational value).

The medical and nurse assessments will be conducted at a University affiliated research and training clinic of the Lek Yuen Health Centre where ongoing community primary care programme for elderly is run with support from the Jockey Club Charities Trust. Cognitive training and physical activity will take place either at the research clinic or at non-governmental organizations (NGOs), (*e.g.*, the Evangelical Lutheran Church Social Service, the Shatin District Community Centre for the Golden Aged, and the Shatin Rhenish Neighbourhood Elderly Centre) where they provide community services for the elderly and have existing working relationships with our team at the Lek Yuen Health Centre based on individual participant's preference.

(iii) Study design

Recruitment

Similar to the studies conducted by two of our investigators who have recruited over 500 older adults in community and primary care settings, participants will be recruited in the community and primary care settings through geriatric day centres and clinics, and elderly social centres/NGOs which provide services for the elderly. We will also recruit from the public general outpatient clinics (GOPCs) and send out invitation letters and posters to community councils in the New-Territory East Cluster (NTEC) for facilitating subject recruitment. All interested participants will undergo screening for cognitive impairment. This screening will consist of the Montreal Cognitive Assessment Hong Kong version (HK-MoCA) conducted by trained research assistants.[24] Potential participants who are screened positive will be further assessed by the research team primary care physicians to determine further eligibility for the current study.

Participants

Inclusion criteria for participants will be: (1) aged between 60 years and 80 years; (2) having Montreal Cognitive Assessment Hong Kong version (HK-MoCA) score within 19-21 after adjusting for years of educational (+1 point if < 6 years) suggesting MCI according to published research;[24] and (3) being physically stable and without life threatening diseases, as assessed by the study physicians.

Exclusion criteria for participants will be: (1) a diagnosis of dementia; (2) concurrent treatment with structured cognitive training, regular physical exercises including Tai Chi or other lifestyle modifications (*e.g.*, diet) although no restrictions will be imposed on leisure activity; (3) a past history of bipolar affective disorder or psychosis; and (4) one or more significant communicative impairments. The use of psychotropic medication; this does NOT constitute an exclusion criterion, but any use of anti-dementia and other psychotropic medications should be kept on a

stable dosage for at least three months before enrolment and also throughout the study. Those who are screened positive for dementia (HK-MoCA ≤ 18) will be referred for management within the outpatient clinics.

Randomization, concealment and blinding

Computer-generated allocation sequencing will be done in blocks of twelve in each intervention venue to ensure the balanced allocation of participants in each venue. This will be done by a statistician who is independent from the study. The allocation sequence will be kept concealed in sealed opaque envelopes by the statistician and it will be revealed only after the participants have been recruited and completed assessments at baseline. The persons who conduct the assessments or data analyses will be blinded to group allocation.

Intervention description

All included participants will meet the study team at screening, baseline, 6 and 12 months after randomisation for measurements of blood pressure, weight and height (BMI) and hip and waist circumference. All participants will also meet the study clinician (one of the investigators) at screening for a detailed medical history and physical examination. At baseline, all participants will receive oral and written information and health advice on diet, physical and cognitive activities that can improve the vascular health (reduce vascular risk) of participants from the study nurse. Blood samples will be collected at baseline, 6 months and 12 months with results mailed to all participants with written information explaining the clinical significance of the results. Participants will be advised to contact their primary care physicians if needed. The two intervention arms additionally will receive the intervention components described below. The fidelity of the study will be ensured through: 1) random check of 10% of the medical records by the study physician for risk factor modification based on a checklist of risk factors involved in the study to see if all risk factors have been covered; 2) check with at least 3 participants in each class if all 24 forms are taught and learnt for Tai Chi classes; 3) check with a checklist of cognitive activity instructions to make sure instructions are followed as planned. Information of each patient will be complied in one folder, and a checklist of interventional activities will be made for each participant of each of the 3 arms respectively so that the study investigators can check and see if the interventions have been provided and followed as planned. Regular meetings within the project team will be also held to ensure the implementations of the interventions.

1) Risk factor modification group (RFM)

The components of this intervention are modelled after the FINGER trial[16] with modifications (frequency of visits) based on local common public primary care practice. Participants will be assessed by a trained research nurse and will receive an individualized lifestyle modification every 3 months by the nurse during the study period. They will therefore meet the nurse at baseline, 3, 6, 9 and 12 months and the physician at 6 and 12 months for evaluation of laboratory test results, anthropometric measures and cardiovascular and metabolic conditions. The aim is to modify individual risk factors for dementia following an established treatment protocol for cognitive impairment or dementia.[6-11] These risk factors will include suboptimal blood pressure or lipid or glucose control, high body mass index (BMI > 25 kg/m^2), smoking, physical inactivity, and an unhealthy diet. The participants will be given both oral and written information on the importance of reducing these risk factors. The diet goals are the same as the intervention in the FINGER trial, that is, to achieve adequate consumption of fruit and vegetables; low-fat options in meat products; sucrose intake 50 g/day; and consumption of fish in at least two

portions per week. Because there is insufficient evidence for the benefits of using dietary supplements (*e.g.*, vitamins such as the E or the B group) related to cognitive functioning, the aim is to achieve an adequate intake with a balanced diet which has been shown to improve cardiovascular health.[25-27] Motivating participants to make necessary lifestyle changes will be an important part of the meetings with the nurse. Motivational interviewing (Appendix 1), which uses a guide toward change called FRAMES (the acronym stands for Feedback, Responsibility, Advice, Menu Options, Empathy and Self-Efficacy) will be applied to motivate the behavioural changes related to physical activity, dietary intake, smoking behaviour and medication use which have been used locally. With reference to local clinical guidelines such as “Hong Kong Reference Framework for Diabetes Care for Adults in Primary Care Settings” and “Hong Kong Reference Framework for Hypertension Care for Adults in Primary Care Settings” as well as oversea clinical guidelines, the aim is to calculate all participants’ individual cardiovascular risk using the QRISK3 score with a 10-year cardiovascular risk of lower than 10% as the treatment target.[28] The blood samples taken will include: for baseline, fasting glucose, fasting lipid profile, HbA1C, serum folate, vitamin B12, serum calcium, liver and renal function, electrolytes, thyroid stimulating hormone (TSH), erythrocyte sedimentation rate (ESR); for follow up visits: fasting glucose, HbA1C and lipid profiles. When initiation or adjustment of pharmacologic treatment is needed, participants will be advised to bring a written report written by the study physician/nurse to inform their own primary care physician.

2) Combined mind body physical exercise, cognitive training and risk factor modification group (CPR)

Both cognitive and mind body physical exercise will follow established interventions previously used and evaluated for older adults with cognitive impairment by one of our investigators.[29] The risk factor modification component follows what is described above. Participants will receive a 30-minute session of Tai Chi given by a Tai Chi master or trained physical therapists under the supervision of the team physician. The 24-forms simplified Tai Chi will be used to increase acceptability among elderly. The 24-forms simplified Tai Chi was developed by expert Tai Chi masters in China and is considered to be simple, safe, and effective enough for public education and health promotion.[29] The introduction course for Tai Chi will last for three times a week over 12 weeks. Each session will last for 30 minutes. After 12 weeks of Tai Chi training, participants will be advised to practice following a video or an audio recording in individual or group either in elderly centres or in their neighbourhoods to maintain the practices. The intervention will be conducted in groups at the neighbourhoods, the NGOs or the research clinic. For cognitive training, cognitive activities commonly endorsed by Chinese older adults according to lifestyle activity reference list previously developed by focused group discussions and survey of cognitively healthy Chinese older adults in Hong Kong[30] will be arranged. These cognitive activities are leisure activities with consensus of higher demands on cognitive efforts, such as reading books, newspapers, or magazines; using computers or surfing the internet; playing board games; playing mah-jong; playing card games; participating in forums or discussions; writing; calligraphy; painting; handicraft such as knitting and needlework; and playing a musical instrument. Three one-hour group sessions will be arranged for any week. Our previous study showed that these cognitive activities can protect cognitive decline over time among the elderly with MCI.[19] Strategies to ensure compliance of Tai Chi exercise after the 12-week class and also cognitive activities will be applied, such as discussions with physical trainer of future exercise time and location and possible group exercise, and also discussion with nurse of preferences, possible barriers and facilitators in doing the

exercise and cognitive activities during the clinical visits or assessments if the compliance is suboptimal. A refresh course will also be provided once a month after the class.

3) Heath Advice (HA) control

As mentioned above, participants in this group will receive health advice in the form of booklets from a nurse focusing on healthy diet and physical, cognitive, and social activities beneficial for management of risk factors, cognition decline and disability prevention at baseline, similar to what is often done in primary care. Blood sample results will be mailed to participants every 6 months and advised to contact their regular physician if needed. They will also meet the study clinician at screening.

The fidelity of interventions will be ensured through the following: 1) random check of 10% of the medical records by the study physician for risk factor modification based on a checklist of risk factors involved in the study to see if all risk factors have been covered; 2) check with at least 3 participants in each class if all 24 forms are taught and learnt for Tai Chi classes; 3) check with a checklist of cognitive activity instructions to make sure instructions are followed as planned. Information of each patient will be complied in one folder, and a checklist of interventional activities will be made for each participant of each of the 3 arms respectively so that the study investigators can check and see if the interventions have been provided and followed as planned. Regular meetings within the project team will be also held to ensure the implementations of the interventions.

Outcomes

Assessments will be conducted at baseline, 6, 12 and 15 months post baseline. Basic demographics will be collected at baseline, including age, sex, education, household income adjusted for household size, and marital status.

Primary Outcome

- *The Chinese version of the Alzheimer's Disease Assessment Scale - cognitive subscale (ADAS-Cog).*[31] ADAS-Cog is a widely used standard global cognitive assessment for clinical interventions to record cognitive symptoms with scores ranging from 0 to 70. It includes subtests on episodic memory, agnosia, ideational apraxia, visuospatial construction, orientation and recognition. Higher scores indicate more severe cognitive impairment. The best cut-off score to differentiate between MCI and healthy adults is ≥4 (sensitivity = 0.73, specificity = 0.69, PPV = 0.90, NPV = 0.40), while the best cut-off score to distinguish dementia is ≥12 (sensitivity = 0.86, specificity = 0.89, PPV = 0.99, NPV = 0.32).[32,33]

Secondary Outcomes

- *CDR sum of box.* It gives a clinical rating of everyday functioning through a semi-structured clinical interview. CDR rates cognition and function impairment in six dimensions (memory, orientation, judgment and problem-solving, community affairs, home and hobbies, and personal care). A sum of scores in each dimension results in a global rating ranging from 0 (no dementia), 0.5 (very mild), 1 (mild), 2 (moderate) to 3 (severe dementia).[34,35]

- *The Disability Assessment for Dementia (DAD).* This assesses functional abilities in both the basic and instrumental activities of daily living.[36]

- *Quality of life (EQ5D).* The EuroQoL 5-D Questionnaire, the five-level version (EQ5D)[37] is a standardized short generic instrument to measure the generic quality of life and it has

been validated in Chinese. It consists of five dimensions of health: mobility, ability to self-care, ability to undertake usual activities, pain and discomfort, and anxiety and depression. Higher scores indicate a better quality of life.

- *Depression and anxiety*. Depression and anxiety will be assessed by using the Chinese versions of the Geriatric Depression Scale[38] and the Geriatric Anxiety Scale.[39]

- *Cigarette smoking, alcohol drinking, diet and physical exercise*. Cigarette smoking and alcohol drinking history, frequency and quantity will be measured. Dietary intake of vegetables, fish, and dairy products, will be recorded by the shortened version of the food frequency questionnaire, which has been validated locally.[40,41] The intensity of physical exercise will be quantified based on the revised Borg's scale, which measures the subject's perceived exertion of physical exercise.[42] The type of physical exercise will be classified in accordance with the classification of late-life leisure activities among the elderly Chinese in Hong Kong, which has already been validated and published.

- *Health service utilization*. Health visits, hospitalization, and the use of medications during the study period will be recorded at each time point.

- *Common diseases*. Information about 18 common diseases will be recorded at baseline. These are hypertension, diabetes, high cholesterol, angina/heart attack, stroke, heart failure, chronic bronchitis, asthma, kidney disease, thyroid problems, back problems, arthritis, liver disease, cancer, eczema/psoriasis, gastro-oesophageal disease, migraine headaches, and others.

Feasibility and Data Monitoring

The investigators of this proposal consist of a multidisciplinary team of experts from primary care and family medicine, psycho-geriatrics, nursing, and public health. The PA has an extensive community network for recruitment and is the PI of a large community primary care programme previously funded by the Jockey Club with 1,000 elderly recruited for various bio-psycho-social services in the community through linking these elderly with NGOs for the elderly in the community by using a multidisciplinary team of nurses, social workers and a project coordinator. One of the investigators (Lam, L.C.) also has an extensive network of participant recruitment in Hong Kong and she has conducted both prospective and intervention studies on ageing and dementia with the development of more than 500 participants with various levels of cognitive function in Hong Kong. She is also in charge of the psycho-geriatric team with regular community services of over 30 residential homes and social centres for the elderly. The Evangelical Lutheran Church Social Service, the Shatin Rhenish Neighbourhood Elderly Centre, and the Shatin District Community Centre for the Golden-Aged are in established partnership with the Jockey Club Community Primary Care Programme and they will be sites for recruitment and interventions in this study.

Data monitoring will be done by a biostatistician not being a co-investigator to monitor the quality of the study every 6 months. Regular internal meetings will be held to ensure the study is conducted based on the research plan.

(iv) Data processing and analysis

Data collection and management

Demographic data (sex, age, educational level, and socio-economic status, *e.g.*, household income by household size) will be collected at baseline. All other measures will be collected at baseline, 6, 12 and 15 months by trained RAs who are blinded to group allocation. To increase the response rate at each time point, facilitation will be sought from staff in the participating elderly centres and at least three telephone calls will be made as assessment reminders by research assistants. Data collection will be conducted at either the participating elderly centres or

at the Lek Yuen Health Centre Jockey Club Community Primary Care Programme/Centre. All the data will be locked in a cabinet or saved in pass-word-protected computers to ensure data safety. No personal identity will be revealed in any reports or publications. Only the research team will have the access to the data.

Statistical analysis

Analysis of variance (ANOVA) for continuous variable or chi-square test for categorical variables will be used to compare the differences of baseline variables between groups. We will use linear mixed models (LMM) with random intercept to investigate the significant changes over time, for both primary and secondary outcome measures. The LMM model with an auto regression of order one (AR1) to account for the correlation of patients' repeated measurements over time provides the means to include subjects with incomplete data (missing data at some data time points) and use all available data to assess the treatment effect over time (i.e., trend or group-time interaction). The LMM approach is in line with the intention-to-treat principle, e.g., including all patients for the analysis, regardless whether patients actually received the treatment or subsequent withdrawal or deviation from the protocol. Per protocol analysis or estimation of complier average causal effect (CACE) will be considered. Furthermore, compliance in interventions (risk factor modification, Tai Chi exercise and cognitive activities) and changes in risk factors and lifestyle behaviours measured in the study will be added into the linear mixed models to explore if these are predictors of changes in health outcomes. A p value of less than 0.05 (two sides) will be regarded as statistically significant.

Sample size estimation

A change of three points in ADAS-Cog represents a minimal clinically relevant change.[43] Our previous trial showed that the mean (standard deviation (SD)) of ADAS-Cog score was 11.6 (3.4) at baseline and it was 7.9 (3.6) at 12 months in the cognitive-physical intervention group among participants with MCI. Assuming participants have a similar mean score of 11.6 and a standard deviation (SD) of 3.4 in ADAS-Cog at baseline, it would need 25 participants per arm with a power of 0.9, α=0.05 (two-tailed), and a dropout rate of 25% over 15 months to detect the minimal clinically relevant change in the CPR group when compared to HA control. Assuming at least half of the minimal clinical relevant change (1.5 points in ADAS-Cog) could be seen in the RFM arm over 15 months compared to HA control, as well as in the CPR arm compared to RFM arm, it will need a sample size of 141 per arm (power=0.9, two-sided α=0.05, dropout rate=25%) (total n=423).

[Word Court: 3,808]

e) Existing Facilities:

Office space and office support are available in the School of Public Health and Primary Care at The Chinese University of Hong Kong, as well as at the Jockey Club Primary Care Programme/Centre (4/F Lek Yuen Health Centre). The Jockey Club Primary Care Programme/Centre would provide additional part-time nurse officers who will help with the PCR or NR, as well as additional laptops needed for the cognitive training.

f) Justification of Requirements:

As this is a large-scale trial with three arms where one of the arms has complex interventions (CPR), as well as each participant's assessments will take around 2 hours each time for four times at

baseline, and at 6 and 12 and 15 months, respectively for the older adults with MCI, a duration of three years is needed for project preparation, recruitment, intervention provision and assessments by dividing participants into different batches at different stages.

One full-time research assistant (RA) will be required to coordinate and monitor the overall progress of the project under the close supervision of the study investigators to ensure the trial quality. She/he will also be responsible for recruitment and conducting outcome measures. One part-time Junior research assistants (JRA) will also be employed to support the RA in recruitment, screening and data collection. In addition, one part-time research nurse and Tai Chi masters or physical trainers will be recruited on a part-time basis to provide the nurse-led lifestyle modification sessions and the physical exercise (Tai Chi), respectively. They will be under the supervision of study clinicians to ensure the intervention quality and safety.

Travel allowances, stationary and materials for cognitive training will be needed to conduct the assessments and interventions at different venues.

g) Purpose and Potential:

The aim of this study is to evaluate a pragmatic complex intervention for the elderly with MCI, the prevalence and disease burden of which is expected to increase rapidly in the coming years. Because there is a lack of study or evaluation on effective intervention among this population group in community and primary care settings, this study is urgently needed in Hong Kong and the findings from this study will inform public health practice on a potentially effective intervention and service model to reduce cognitive decline among the elderly with MCI. There is a high potential for the translation of findings into public health practice in community and primary care.

h) Key References:

1. World Health Organization. Dementia: a public health priority. *Alzheimer's Disease International. .* 2012;Geneva: World Health Organization. Accessed February 3, 2016.
2. Alzheimer's Disease International. Policy Brief for Heads of Government. The Global Impact of Dementia 2013–2050. *London, UK, Alzheimer's Disease International. https://www.alz.co.uk/research/GlobalImpactDementia2013.pdf.* 2013;Accessed on Feb 15, 2016.
3. Wimo A, Jönsson L, Bond J, Prince M, Winblad B, International AD. The worldwide economic impact of dementia 2010. *Alzheimer's & dementia.* 2013;9(1):1-11. e13.
4. Chan KY, Wang W, Wu JJ, et al. Epidemiology of Alzheimer's disease and other forms of dementia in China, 1990-2010: a systematic review and analysis. *Lancet.* Jun 8 2013;381(9882):2016-2023.
5. Lam LCW, Tam CWC, Lui VWC, et al. Prevalence of very mild and mild dementia in community-dwelling older Chinese people in Hong Kong. *International Psychogeriatrics.* Feb 2008;20(1):135-148.
6. Deckers K, van Boxtel MPJ, Schiepers OJG, et al. Target risk factors for dementia prevention: a systematic review and Delphi consensus study on the evidence from observational studies. *International journal of geriatric psychiatry.* Mar 2015;30(3):234-246.
7. Stephan BCM, Brayne C. Risk Factors and Screening Methods for Detecting Dementia: A Narrative Review. *J Alzheimers Dis.* 2014;42:S329-S338.

8. Kloppenborg RP, van den Berg E, Kappelle LJ, Biessels GJ. Diabetes and other vascular risk factors for dementia: Which factor matters most? A systematic review. *Eur J Pharmacol.* May 6 2008;585(1):97-108.
9. Patterson C, Feightner J, Garcia A, MacKnight C. General risk factors for dementia: A systematic evidence review. *Alzheimers Dement.* Oct 2007;3(4):341-347.
10. Li JQ, Tan L, Wang HF, et al. Risk factors for predicting progression from mild cognitive impairment to Alzheimer's disease: a systematic review and meta-analysis of cohort studies. *Journal of neurology, neurosurgery, and psychiatry.* May 22 2015.
11. Cooper C, Sommerlad A, Lyketsos CG, Livingston G. Modifiable predictors of dementia in mild cognitive impairment: a systematic review and meta-analysis. *Am J Psychiatry.* Apr 2015;172(4):323-334.
12. Lee AT, Richards M, Chan WC, Chiu HF, Lee RS, Lam LC. Intensity and Types of Physical Exercise in Relation to Dementia Risk Reduction in Community-Living Older Adults. *Journal of the American Medical Directors Association.* Oct 1 2015;16(10):899 e891-897.
13. Daviglus M, Bell C, Berrettini W, et al. National Institutesof Health State-of-the-Science Conference Statement: Preventing Alzheimer’s Disease and Cognitive Decline. . *NIH Consens State Sci Statements.* 2010;Apr 26–28(27(4)):1–30.
14. Olanrewaju O, Clare L, Barnes L, Brayne C. A multimodal approach to dementia prevention: A report from the Cambridge Institute of Public Health. *Alzheimer's & Dementia: Translational Research & Clinical Interventions.* 2015;1(3):151-156.
15. Richard E, van Charante EPM, van Gool WA. Vascular Risk Factors as Treatment Target to Prevent Cognitive Decline. *J Alzheimers Dis.* 2012;32(3):733-740.
16. Ngandu T, Lehtisalo J, Solomon A, et al. A 2 year multidomain intervention of diet, exercise, cognitive training, and vascular risk monitoring versus control to prevent cognitive decline in at-risk elderly people (FINGER): a randomised controlled trial. *Lancet.* Jun 6 2015;385(9984):2255-2263.
17. Mangialasche F, Kivipelto M, Solomon A, Fratiglioni L. Dementia prevention: current epidemiological evidence and future perspective. *Alzheimer's research & therapy.* 2012;4(1):6.
18. Vellas B, Carrie I, Gillette-Guyonnet S, et al. Mapt Study: A Multidomain Approach for Preventing Alzheimer's Disease: Design and Baseline Data. *J Prev Alzheimers Dis.* Jun 2014;1(1):13-22.
19. Lam LC, Chan WC, Leung T, Fung AW, Leung EM. Would older adults with mild cognitive impairment adhere to and benefit from a structured lifestyle activity intervention to enhance cognition?: a cluster randomized controlled trial. *Plos One.* 2015;10(3):e0118173.
20. Hospital Authority Hong Kong SAR. AOM Paper No. 801 - Third Update on the Programmes under Chronic Disease Management and Way Forward. 2011(http://www.ha.org.hk/haho/ho/cad_bnc/aom-p801.pdf):Accessed on Feb 15, 2016.
21. Department of Health Hong Kong SAR. Primary Care Initiatives. Hospital Authority Projects. *http://www.pco.gov.hk/english/initiatives/projects.html.* 2016;Accessed on Feb 15, 2016.
22. Hong Kong SAR. The 2016 Policy Address. Innovate for the Economy Improve Livelihood Foster Harmony Share Prosperity. *http://www.policyaddress.gov.hk/2016/eng/.* 2016;Public Healthcare

Services (227):Accessed on Feb 15, 2016.

23. Vellas B, Oustric S. Alzheimer's Preventive Approaches and Cognitive Monitoring Must Be Integrated Into the Primary Care Setting. *Journal of the American Medical Directors Association.* Nov 2014;15(11):783-785.
24. Yeung P, Wong L, Chan C, Leung J, Yung C. A validation study of the Hong Kong version of Montreal Cognitive Assessment (HK-MoCA) in Chinese older adults in Hong Kong. *Hong Kong Med J.* 2014;20(6):504-510.
25. Kwok TC, Lam LC, Sea MM, Goggins W, Woo J. A randomized controlled trial of dietetic interventions to prevent cognitive decline in old age hostel residents. *Eur J Clin Nutr.* Oct 2012;66(10):1135-1140.
26. Kivipelto M, Solomon A, Ahtiluoto S, et al. The Finnish Geriatric Intervention Study to Prevent Cognitive Impairment and Disability (FINGER): study design and progress. *Alzheimer's & dementia : the journal of the Alzheimer's Association.* Nov 2013;9(6):657-665.
27. Lehert P, Villaseca P, Hogervorst E, Maki PM, Henderson VW. Individually modifiable risk factors to ameliorate cognitive aging: a systematic review and meta-analysis. *Climacteric.* 2015;18(5):678-689.
28. National Institute for Health and Care Excellence (NICE) UK. Cardiovascular disease: risk assessment and reduction, including lipid modification (Clinical guideline [CG181]). Published in July 2014. Last updated in September 2016. https://wwwniceorguk/guidance/cg181. Accessed on December 17, 2018.
29. Lam LC, Chau RC, Wong BM, et al. A 1-year randomized controlled trial comparing mind body exercise (Tai Chi) with stretching and toning exercise on cognitive function in older Chinese adults at risk of cognitive decline. *Journal of the American Medical Directors Association.* Jul 2012;13(6):568 e515-520.
30. Leung GTY, Leung KF, Lam LCW. Classification of late-life leisure activities among elderly Chinese in Hong Kong. *East Asian Archives of Psychiatry.* 2011;21(3):123.
31. Chu LW, Chiu KC, Hui SL, Yu GK, Tsui WJ, Lee PW. The reliability and validity of the Alzheimer's Disease Assessment Scale Cognitive Subscale (ADAS-Cog) among the elderly Chinese in Hong Kong. *Ann Acad Med Singapore.* Jul 2000;29(4):474-485.
32. Monllau A, Pena-Casanova J, Blesa R, et al. Diagnostic value and functional correlations of the ADAS-Cog scale in Alzheimer's disease: data on NORMACODEM project. *Neurologia (Barcelona, Spain).* 2007;22(8):493.
33. Zainal NH, Silva E, Lim LL, Kandiah N. Psychometric Properties of Alzheimer’s Disease Assessment Scale-Cognitive Subscale for Mild Cognitive Impairment and Mild Alzheimer’s Disease Patients in an Asian Context. *Ann Acad Med Singapore.* 2016;45:273-283.
34. Morris JC. Clinical dementia rating: a reliable and valid diagnostic and staging measure for dementia of the Alzheimer type. *Int Psychogeriatr.* 1997;9 Suppl 1:173-176; discussion 177-178.
35. Hughes CP, Berg L, Danziger WL, Coben LA, Martin RL. A new clinical scale for the staging of dementia. *Br J Psychiatry.* Jun 1982;140:566-572.
36. Mok CC, Siu AM, Chan WC, Yeung KM, Pan PC, Li SW. Functional disabilities profile of chinese

elderly people with Alzheimer's disease - a validation study on the chinese version of the disability assessment for dementia. *Dementia and geriatric cognitive disorders.* 2005;20(2-3):112-119.

37. Luo N, Chew LH, Fong KY, et al. Validity and reliability of the EQ-5D self-report questionnaire in Chinese-speaking patients with rheumatic diseases in Singapore. *ANNALS-ACADEMY OF MEDICINE SINGAPORE.* 2003;32(5):685-690.
38. Chiu HF, Lee HC, Wing YK, Kwong PK, Leung CM, Chung DW. Reliability, validity and structure of the Chinese Geriatric Depression Scale in a Hong Kong context: a preliminary report. *Singapore Med J.* Oct 1994;35(5):477-480.
39. Lin X, Haralambous B, Pachana NA, et al. Screening for depression and anxiety among older Chinese immigrants living in Western countries: The use of the Geriatric Depression Scale (GDS) and the Geriatric Anxiety Inventory (GAI). *Asia Pac Psychiatry.* Mar 2016;8(1):32-43.
40. Yu R, Woo J, Chan R, et al. Relationship between dietary intake and the development of type 2 diabetes in a Chinese population: the Hong Kong Dietary Survey. *Public Health Nutr.* Jul 2011;14(7):1133-1141.
41. Sea MMM, Woo J, Tong PCY, Chow CC, Chan JCN. Associations between food variety and body fatness in Hong Kong Chinese adults. *J Am Coll Nutr.* Oct 2004;23(5):404-413.
42. Borg GA. Psychophysical bases of perceived exertion. *Med Sci Sports Exerc.* 1982;14(5):377-381.
43. Schrag A, Schott JM, Alzheimer's Disease Neuroimaging Initiative. What is the clinically relevant change on the ADAS-Cog? *J Neurol Neurosur Ps.* Feb 2012;83(2):171-173.
